# Heart failure with preserved ejection fraction in women: the Dutch Queen of Hearts program

**DOI:** 10.1007/s12471-014-0613-1

**Published:** 2015-01-23

**Authors:** H. den Ruijter, G. Pasterkamp, F. H. Rutten, C. S. P. Lam, C. Chi, K. H. Tan, A. J. van Zonneveld, M. Spaanderman, D. P. V. de Kleijn

**Affiliations:** 1 Experimental Cardiology Laboratory, University Medical Center, Utrecht, the Netherlands; 2 Julius Center for Health Sciences and Primary Care, University Medical Center Utrecht, Utrecht, the Netherlands; 3 National University Health System, Singapore, Singapore; 4 Women’s Centre, Department of Obstetrics & Gynaecology, National University Singapore, Singapore, Singapore; 5 Division of Obstetrics & Gynaecology, KK Women’s and Children Hospital, Singapore, Singapore; 6 Department of Nephrology and the Einthoven Laboratory for Experimental Vascular Medicine, Leiden University Medical Center, Leiden, the Netherlands; 7 Department of Obstetrics & Gynaecology, Maastricht University Medical Center, Maastricht, the Netherlands; 8 Surgery, National University Singapore, Singapore, Singapore; 9 Cardiovascular Research Institute, NUHCS, NUHS, Singapore, Singapore; 10 Interuniversity Cardiology Institute of the Netherlands, Moreelsepark 1, 3511 EP Utrecht, the Netherlands

**Keywords:** Heart failure with preserved ejection fraction, Gender

## Abstract

Heart failure (HF) poses a heavy burden on patients, their families and society. The syndrome of HF comes in two types: with reduced ejection fraction (HFrEF) and preserved ejection fraction (HFpEF). The latter is on the increase and predominantly present in women, especially the older ones. There is an urgent need for mortality-reducing drugs in HFpEF, a disease affecting around 5 % of those aged 65 years and over. HFpEF develops in patients with risk factors and comorbidities such as obesity, hypertension, diabetes, COPD, but also preeclampsia. These conditions are likely to drive microvascular disease with involvement of the coronary microvasculature, which may eventually evolve into HFpEF. Currently, the diagnosis of HFPEF relies mainly on echocardiography. There are no biomarkers that can help diagnose female microvascular disease or facilitate the diagnosis of (early stages of) HFpEF. Recently a Dutch consortium was initiated, Queen of Hearts, with support from the Netherlands Heart Foundation, with the aim to discover and validate biomarkers for diastolic dysfunction and HFpEF in women. These biomarkers come from innovative blood-derived sources such as extracellular vesicles and circulating cells. Within the Queen of Hearts consortium, we will pursue female biomarkers that have the potential for further evolution in assays with point of care capabilities. As a spin-off, the consortium will gain knowledge on gender-specific pathology of HFpEF, possibly opening up novel treatment options.

## Women and heart failure with preserved ejection fraction

Cardiovascular disease (CVD) is the number one killer among women worldwide. Controversially, women have been underrepresented in most clinical trials on cardiovascular disease. The most striking differences in the prevalence of CVD between men and women are visualised in the syndrome of heart failure (HF).

Among older patients in developed countries, HF probably represents the greatest health burden with an estimated 23 million people with HF worldwide, with nearly 50 % of them women [1]. HF is a clinical syndrome characterised by symptoms and signs of volume overload and cardiac adaptation, where cardiac dysfunction is responsible for failure of the heart to supply adequate peripheral oxygen delivery to meet the requirements of metabolising tissues. Without myocardial damage in the history, the lifetime risk of HF at age 40 is estimated to be 1 in 6 for women, and 1 to 9 in men [1]. Most studies agree that women suffer from a worse quality of life when HF has been diagnosed [2]. Despite this, survival for HF seems to be better for women [3] underlining the gender differences in development and progression of HF.

The diagnosis of HF is based on suggestive symptoms and signs and structural or functional abnormalities on echocardiography [4, 5]. Patients with HF and a preserved ejection fraction (HFpEF) have similar symptoms and signs to those with reduced ejection fraction (HFrEF). Around 50 % of the patients with HF suffer from HFpEF, also referred to as diastolic HF. The aetiology of HFrEF and HFpEF differs, and prognostically beneficial therapy for HFpEF is lacking, which is in sharp contrast to HFrEF.

HFpEF is associated with diastolic left ventricular dysfunction that involves reduced left ventricular relaxation and increased left ventricular stiffness with a relatively normal ejection fraction of 50 % or more [6]. A consistent and unexplained finding among population-based studies is that women outnumber men in the syndrome of HFpEF with an impressive 2:1 ratio.

Natriuretic peptides are useful biomarkers with added diagnostic value on top of clinical assessment [7], although with much better negative predictive values (exclusion of HF) than positive predictive values (‘confirming’ HF) [4]. Importantly, however, in HFpEF natriuretic peptide levels can be at normal levels, especially in the early phases of the disease, when the patient has not exercised in the last couple of days, and in the absence of signs of water and salt retention. Screening studies showed that unrecognised HF is common in high-risk groups such as older community-dwelling individuals with chronic obstructive pulmonary disease (COPD) and diabetes type 2, with prevalence rates of undetected HF of 20.5 and 27.7 %, respectively [8, 9]. In those with COPD aged 65 years and over and novel screen-detected heart failure, 50 % had HFpEF. In those with type 2 diabetes aged 60 years and over with screen-detected HF, 83 % had HFpEF. Interestingly, the prevalence of unrecognised HFpEF in patients with type 2 diabetes was much higher in women than in men (28.0 vs. 18.4 %), and this was in all age strata [9].

The prevalence of HFpEF is around 3–5 % in the open population aged 65 years and over, and 4–6 % in men and 8–10 % in women for individuals of 80 years and older [10]. Due to the ageing (female) population, HFpEF is a growing epidemic with poor treatment options resulting in more elderly women living with disabling HFpEF [6].

## Comorbidities

Comorbidities have been strongly associated with HF but differ between HFrEF and HFpEF. In a predominantly male HF cohort it was shown that there was a higher non-cardiac comorbidity burden in HFpEF patients compared with HFrEF patients [11].

Differences in prevalence of comorbidities also exist between men and women with HFpEF. Women are generally older and have a higher likelihood of having diabetes, systemic hypertension and systolic blood pressure with a similar body mass index to men [12].

### Diabetes type 2

Diabetes is one of the main risk factors for developing HFpEF. This is evident in Western society but even more eminent in Asian people. The prevalence of diabetes is rapidly rising which is likely to push up the incidence of HFpEF in the coming decades. Astounding prevalence rates of diastolic dysfunction between 27 and 70 % have been reported in the growing diabetics [13]. As already mentioned, the prevalence of unknown HFpEF in patients with type 2 diabetes and aged 60 years or over was clearly higher in women than men [9].

### Hypertension and preeclampsia

Hypertension is a major risk factor for HF as it increases cardiac workload. Hypertension is more prevalent in HFpEF than in HFrEF [11]. Systemic hypertension is very common (83 %) and often severe in HFpEF patients [13]. The Framingham Heart Study already identified hypertension as the greatest population attributable risk for the development of HF with 39 % in men and 59 % in women [14]. Furthermore, among those with hypertension, diabetes poses an additional risk for the development of HF, which is higher in women (HR 3.57 95 % CI (2.59–4.94) than men (HR 1.78 95 % CI (1.23–2.59)).

Preeclampsia is the occurrence of both hypertension and proteinuria during pregnancy and is associated with the development of hypertension, diabetes type 2 and obesity later in life. Although the exact pathophysiology of preeclampsia is unclear, it is already known that it can result in endothelial dysfunction during the pregnancy [15]. Preeclampsia is a typical female risk factor for the development of HFpEF which has been underexposed.

Preeclampsia can already be associated with diastolic dysfunction during pregnancy, but also 1 year after pregnancy. In a small cohort of women with preterm preeclampsia, diastolic dysfunction persisted up to 1 year postpartum in 51 % of the women [16]. In the women with term preeclampsia, diastolic dysfunction decreased from 40 % during pregnancy to 16 % after 1 year post-partum, while in controls this was approximately 5 % at both time points. Moreover, the risk of developing hypertension in women with preeclampsia was higher 2 years postpartum than in women without preeclampsia. Preliminary data suggest that preeclampsia is associated with placenta dysfunction and is a trigger for accelerated microvascular endothelial dysfunction [17].

## Microvascular disease as underlying cause of HFpEF

Diabetes, hypertension and preeclampsia have in common that they accelerate systemic and coronary microvascular dysfunction, which is highly associated with the development of HFpEF. This relation seems to be stronger in women. In contrast, HFrEF is typically myocardial infarction with eccentric remodelling as a result [4]. A dilated left ventricle, a reduction in stroke volume and left ventricular ejection fraction can be found.

HFpEF seems thus to be a different phenotype within the HF spectrum. According to a recently postulated paradigm, HFpEF develops from a ‘systemic proinflammatory state’ induced by certain comorbidities [17].

Comorbidities such as diabetes, obesity, COPD and preeclampsia can induce a systemic inflammatory state and subsequent systemic microvascular endothelial dysfunction [17]. The proinflammatory state causes microvascular endothelial dysfunction due to reduced bioavailability of nitric oxide and concentric remodelling of the left ventricle. In sharp contrast with HFrEF, well-established evidence-based heart failure therapies such as ACE inhibitors, angiotensin receptor blockers, mineralocorticoid receptor antagonists, and beta-blockers did not significantly improve the prognosis in HFpEF. More ‘upstream’ treatment of HFpEF seems to be a better option, but for that the pathways to diastolic dysfunction and finally HFpEF should be more clearly unravelled.

## The Queen of Hearts concept

The Queen of Hearts program (http://www.queen-of-hearts.eu) hypothesises that comorbidities such as diabetes, COPD, and preeclampsia share a common pathway to the development of HFpEF from which gender-specific biomarkers in women can be deduced. By using follow-up echocardiographic data in women who have experienced preeclampsia, we hope to detect novel biomarkers that could be helpful in clinical practice for individual risk stratification and diagnostic and prognostic prediction.

**Fig. 1:**
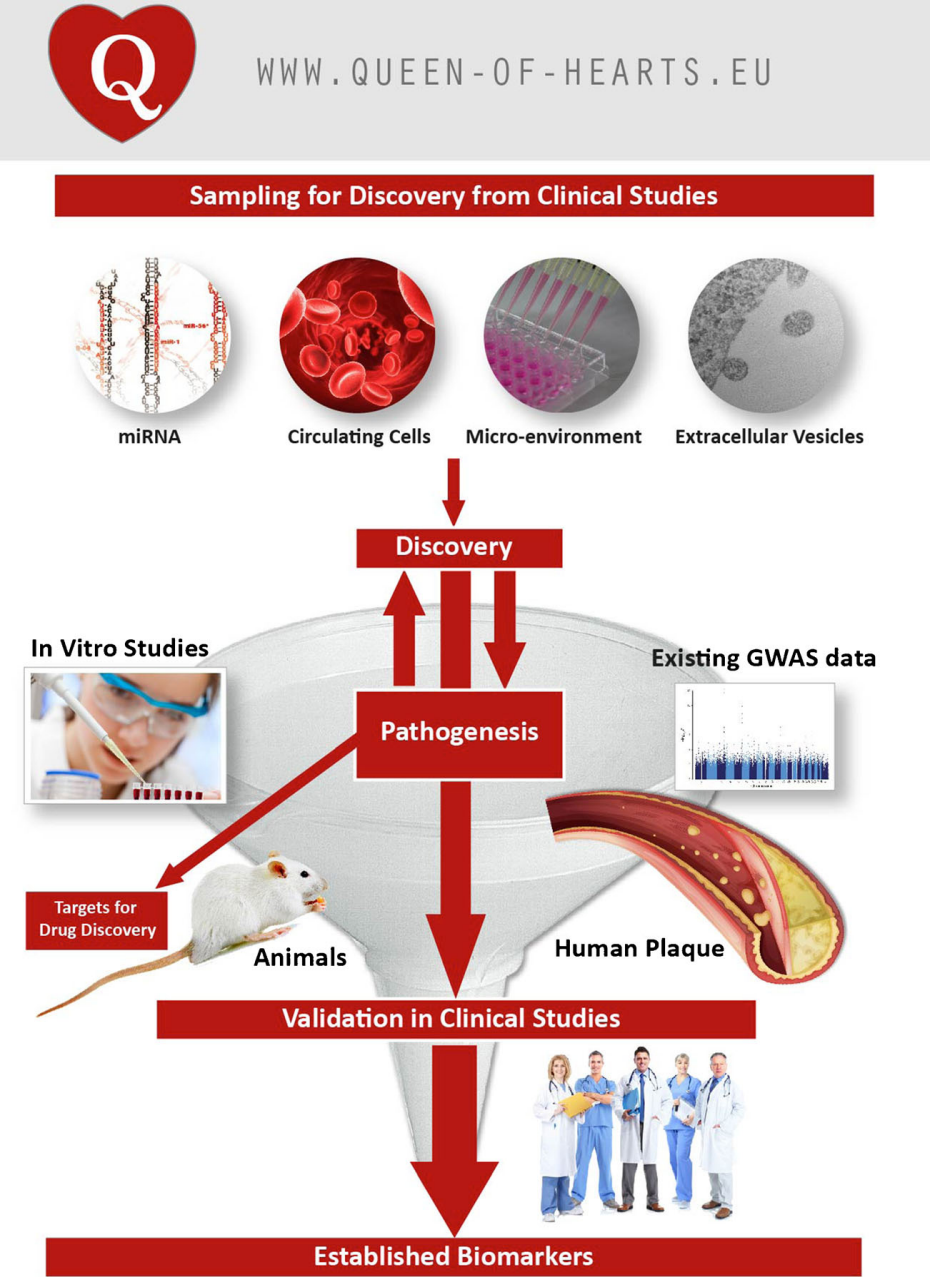
The Queen of Hearts consortium consists of three themes that are interconnected: Discovery of biomarkers, Pathogenesis and Validation in clinical studies. Copied with permission from www.queen-of-hearts.eu

The Queen of Hearts program (Fig. 1) aims to discover novel biomarkers from blood-derived sources (miRNA, extracellular vesicles, micro-environment, circulating cells) for sex-specific biomarker discovery based on microvascular pathology. This approach will also help in understanding the underlying pathogenesis of diastolic dysfunction and HFpEF. The ultimate goal is to facilitate female-specific diagnostic and prognostic blood-based tests for diastolic dysfunction and HFpEF in the clinical setting.

## Global and future perspectives

From the WHO statistics report 2012 (http://www.who.int/gho/publications/world_health_statistics/2012/en/) it is clear that diabetes and hypertension are on the increase worldwide, also affecting low income countries. Asia and Africa are overtaking the Western world and as a result cardiovascular diseases, including heart failure, are on the increase in these parts of the world.

### HFpEF in Asia

In contrast to the wealth of data regarding HFpEF in Western populations, data are limited in Asian populations. The true prevalence of HFpEF in Asian communities is unknown, and may vary in different regions of Asia. In Singapore, the proportion of HF patients with HFpEF has been estimated at 22 % in a single-centre study [18]—this is a lower proportion than that seen in Western cohorts and remains to be confirmed in larger prospective studies. One such study is the ongoing Singapore Heart Failure Outcomes and Phenotypes (SHOP) study [19]. Data in a subset of 50 Asian patients with HFpEF from the SHOP cohort showed that these were more predominantly elderly (mean age 69 years) hypertensive women with a higher mean body mass index compared with patients with HFrEF or community-based controls without HF. Furthermore, compared with age-matched community-based controls without heart failure, patients with HFpEF had higher median levels of growth differentiation factor 15, a member of the transforming growth factor-beta cytokine superfamily and marker of cell injury and inflammation (540 pg/ml vs 2529 pg/ml). This is consistent with a systemic inflammatory response in Asian patients with HFpEF. Larger prospective studies are underway across Asia to further define the Asian phenotype of HF [20]. In addition, recognising the close link between pregnancy-associated diseases and development of cardiovascular diseases including HFpEF in women, a combined clinical program integrating Obstetrics & Gynaecology and Cardiology has been set up in Singapore. The combined study of these women from both disciplines provides the potential to elucidate shared novel pathways between preeclampsia and HFpEF.

Next to these initiatives, Singapore has a comparable health standard to the Netherlands and is inhabited by three Asian ethnicities. The Queen of Hearts program aims to strongly collaborate with this Asian country. Initiatives are being taken for exchange of people and validation of new markers in Singaporean cohorts.

### Future perspectives

The number of older women with a history of hypertension and diabetes will increase worldwide and thus, early risk stratification and early and accurate diagnosis of HFpEF with the help of novel blood markers could help to identify high-risk patients, and provide the opportunity for novel treatment options.

The Queen of Hearts program anticipates to discover novel proteins and miRNAs that may improve risk stratification, early diagnosis and provide novel treatment targets for HFpEF, especially in well-established high-risk groups such as women with diabetes or a history of preeclampsia. We will collaborate with international cohorts of heart failure and preeclampsia in Singapore and Asia.

The consortium will improve the knowledge on gender-specific pathology of HFpEF.
